# Supplementary material for: A novel method for assessing climate change impacts in ecotron experiments
Source: Int J Biometeorol. 2020 Jul 27;64(10):1709–27. doi: 10.1007/s00484-020-01951-8 (PMC7481170; doi:10.1007/s00484-020-01951-8)
Supplement: Supplementary file 1 — (PDF 68.3 MB) [file 484_2020_1951_MOESM1_ESM.pdf]

Supplementary Material for "A novel method for assessing climate change impacts in ecotron experiments"

Inne Vanderkelen[1], Jakob Zschleischler[2,3], Lukas Gudmundsson[4], Klaus Keuler[5], Francois Rineau[6], Natalie Beenaerts[6], Jaco Vangronsveld[6,7], Sara Vicca[8], and Wim Thiery[1,4]

[1]Department of Hydrology and Hydraulic Engineering, Vrije Universiteit Brussel, Brussels, Belgium
[2]Climate and Environmental Physics, University of Bern, Bern, Switzerland
[3]Oeschger Center for Climate Change Research, University of Bern, Bern, Switzerland
[4]Institute for Atmospheric and Climate Science, ETH Zurich, Zurich, Switzerland
[5]Department of Environmental Meteorology, Brandenburg University of Technology Cottbus-Senftenberg, Cottbus, Germany
[6]Centre for Environmental Sciences, UHasselt, Hasselt, Belgium
[7]Department of Plant Physiology, Faculty of Biology and Biotechnology, Maria Curie-Sklodowska University, Lublin, Poland
[8]Department of Biology, University of Antwerp, Wilrijk, Belgium.

Contents of this file

This supplementary file contains 8 supplementary figures providing background information to the main manuscript.

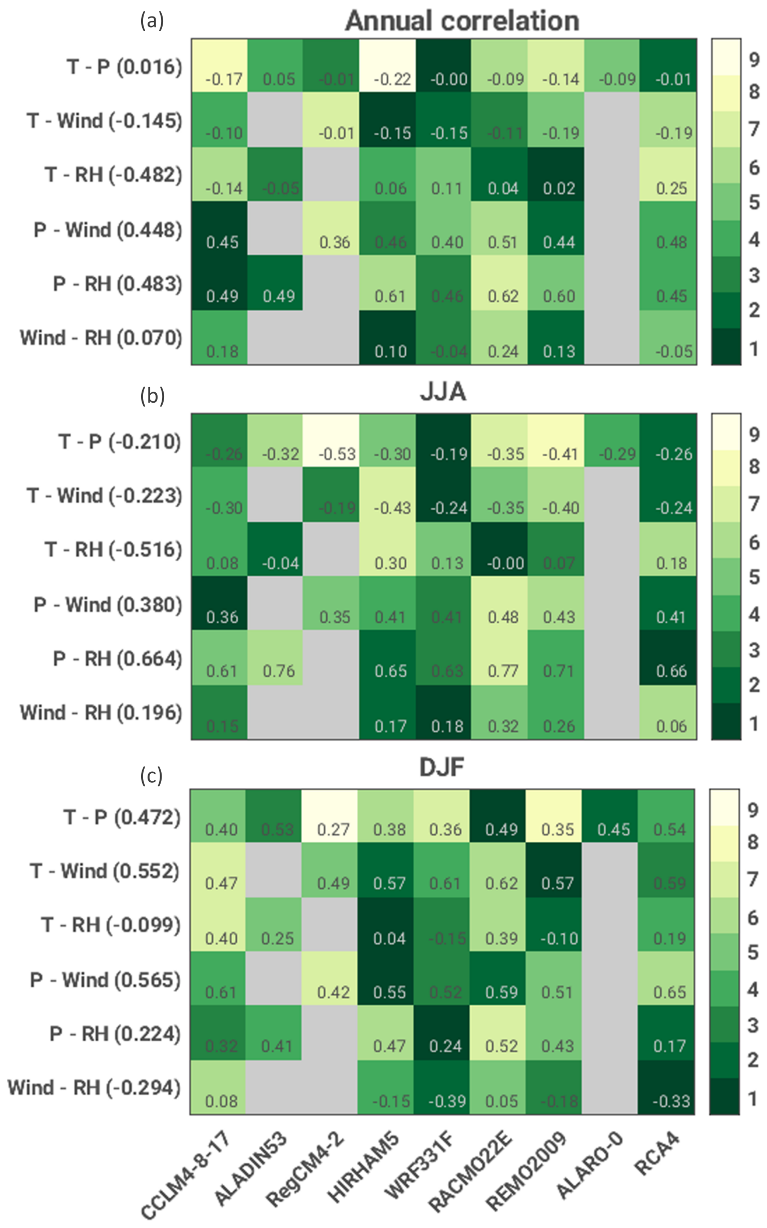

Figure A1. Correlations for the reanalysis downscalings (1990-2008): Annual correlations (a), correlations in June, July and August (JJA; b) and correlations in December, January and February (DJF; c). T stands for air temperature, P for precipitation and RH for relative humidity. The values in the y-axis labels are the observed correlations, and the other values correlations between the simulated variables. Rankings are from 1-best to 9-worst.

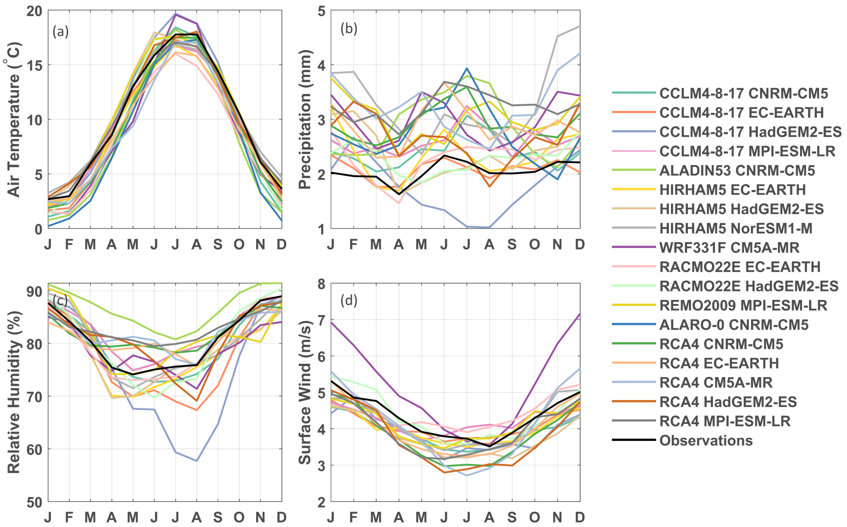

Figure A2. Seasonal cycle of the GCM downscalings for mean air temperature (a), mean daily precipitation (b), mean relative humidity (c) and mean surface wind speed (d).

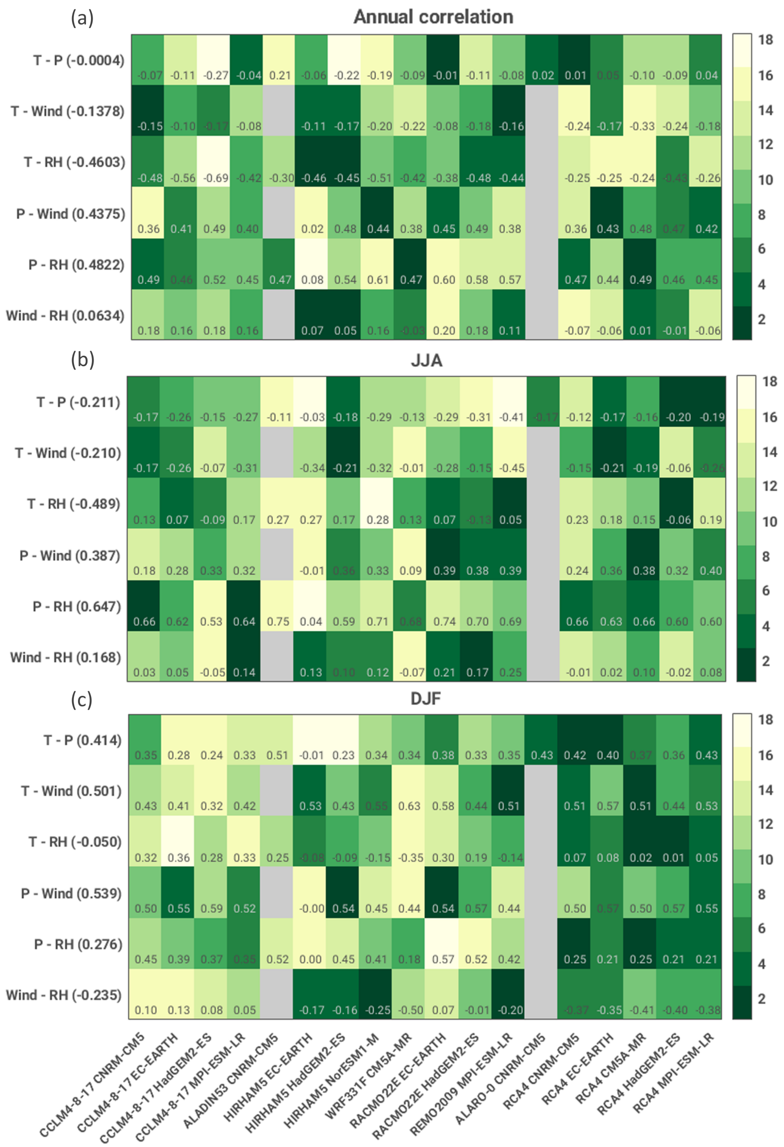

Figure A3. Correlations for the GCM downscalings (1951-2005): Annual correlations (a), correlations in June, July and August (JJA; b) and correlations in December, January and February (DJF; c). T stands for air temperature, P for precipitation and RH for relative humidity. The values in the y-axis labels are the observed correlations, and the other values correlations between the simulated variables. Rankings are from 1-best to 9-worst.

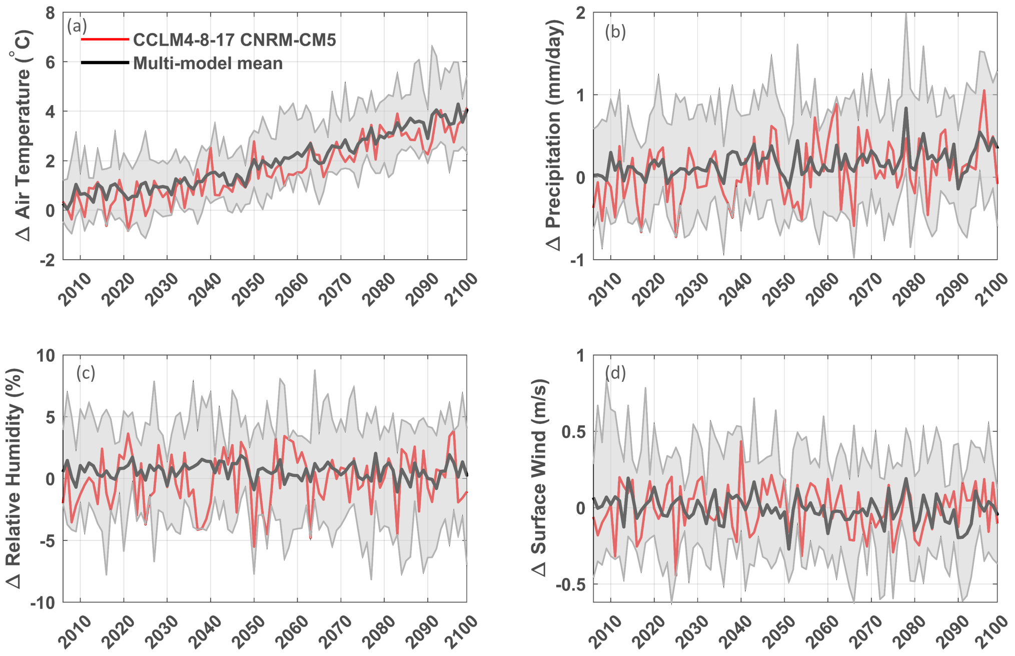

Figure A4. Anomalies for the CCLM4-8-17 CNRM-CM5 simulation following RCP 8.5 at the ecotron site for mean air temperature (a), mean daily precipitation (b), mean relative humidity (c) and mean surface wind speed (d). The reference period is 1977 to 2006, the anomalies of the CCLM4-8-17 CNRM-CM5 simulation are calculated compared to its own values in the reference period. In gray the envelope of all EURO-CORDEX RCP8.5 simulations is showed (See Fig. 8).

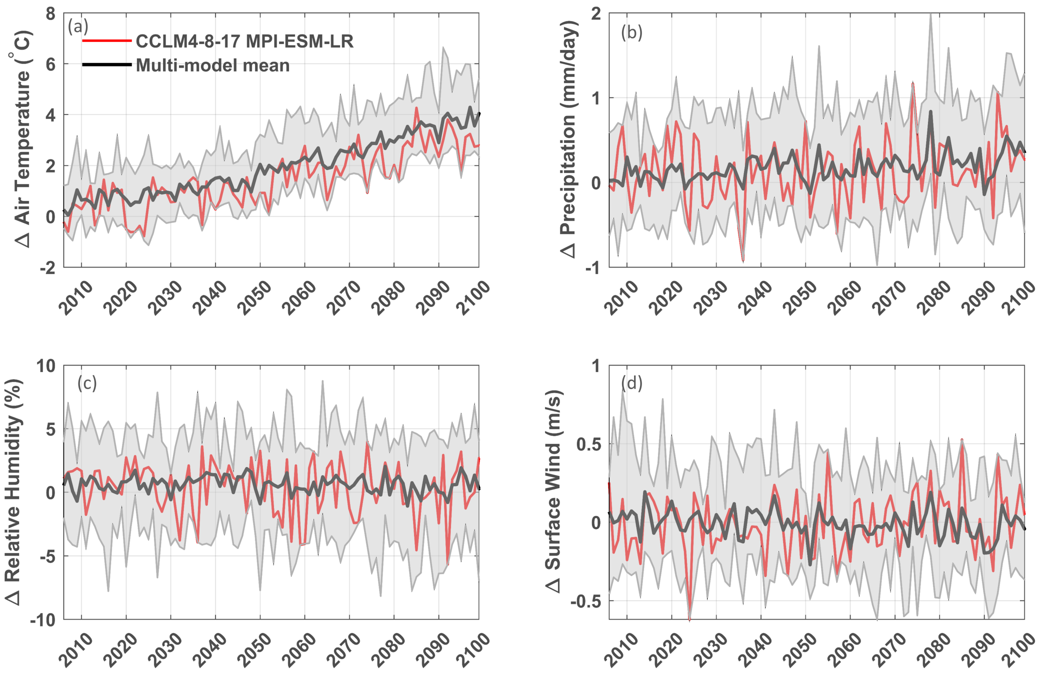

Figure A5. Same as Fig. A4, but now for CCLM4-8-17 MPI-ESM-LR.

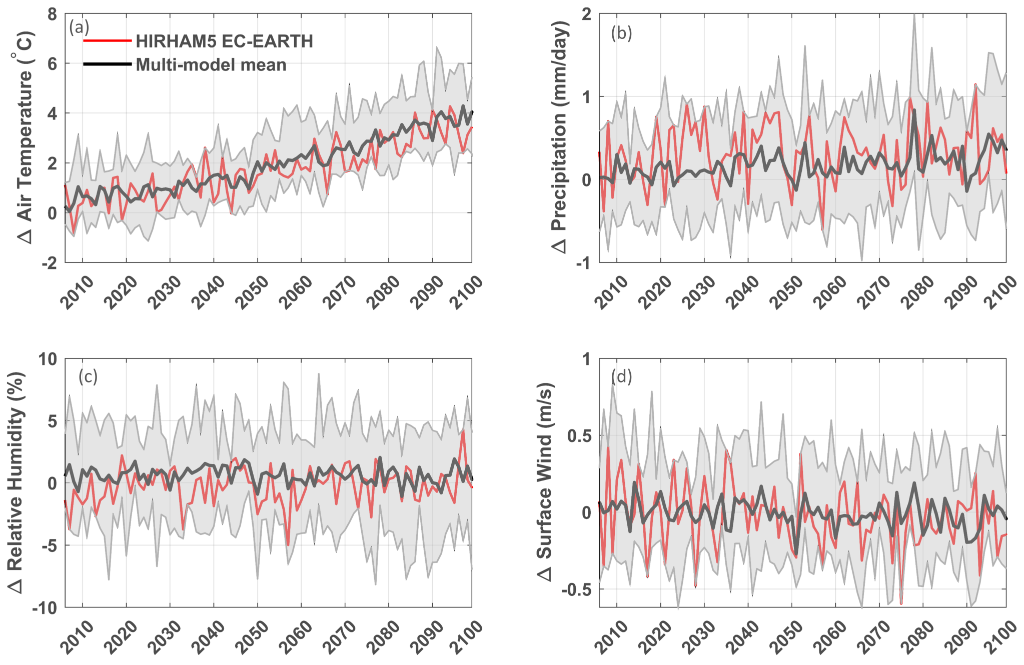

Figure A6. Same as Fig. A4, but now for HIRHAM5 EC-EARTH.

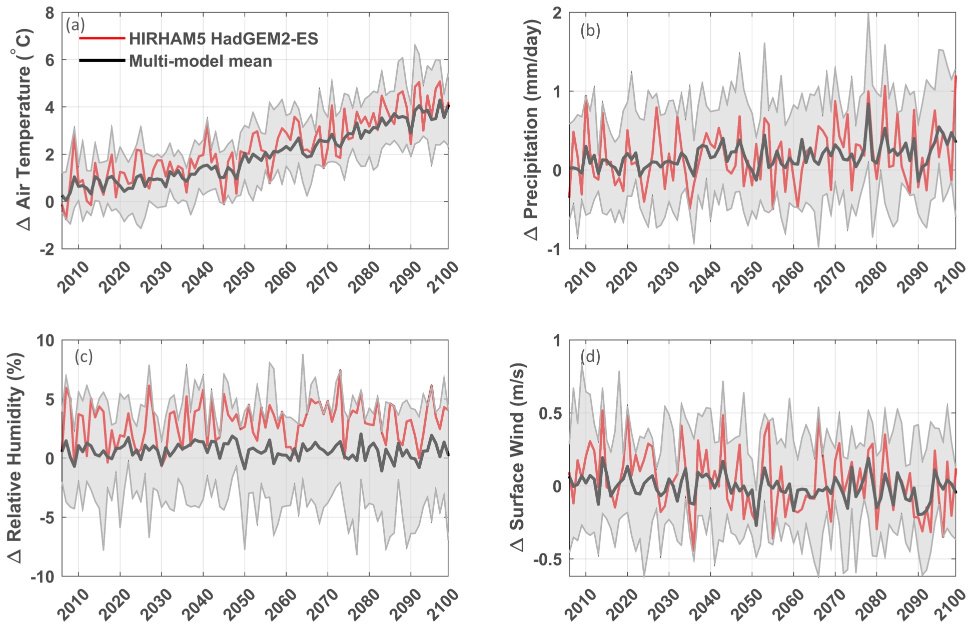

Figure A7. Same as Fig. A4, but now for HIRHAM5 HadGEM2-ES.

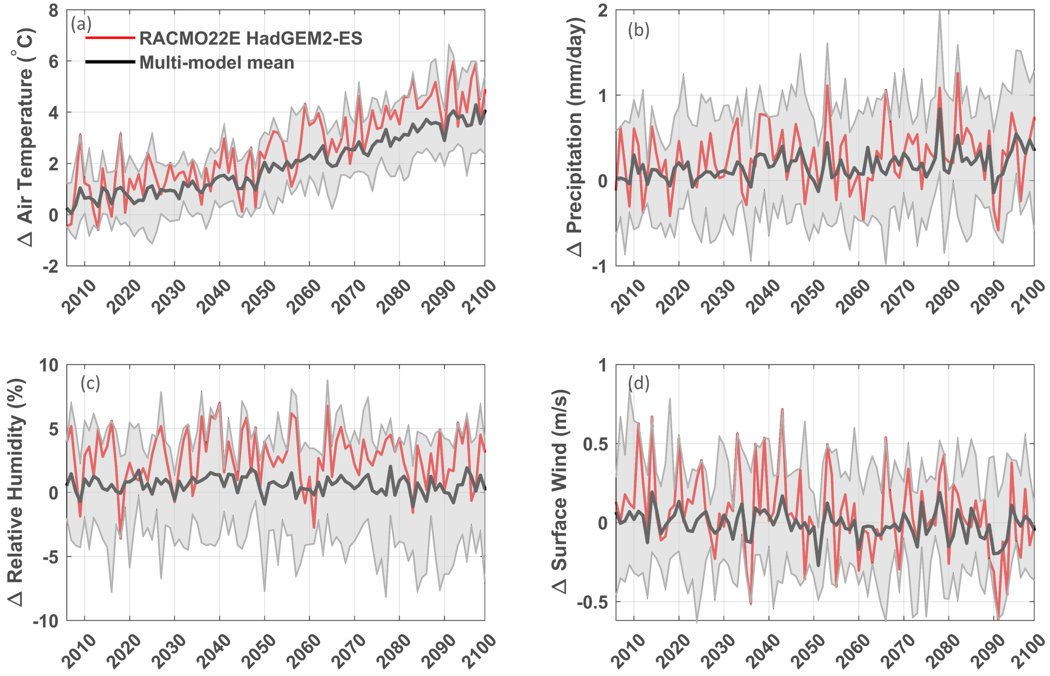

Figure A8. Same as Fig. A4, but now for RACMO22E HadGEM2-ES.
